# Functional capacity and health status in patient with chronic obstructive pulmonary disease

**DOI:** 10.1080/07853890.2021.1896586

**Published:** 2021-09-28

**Authors:** Ângela Maria Pereira, Ernesto Pereira, Helena Santa-Clara

**Affiliations:** a Department of Physiotherapy, Escola Superior de Saúde Egas Moniz (ESSEM), Egas Moniz Cooperativa de Ensino Superior, Monte da Caparica, Portugal; b Centro de Investigação Interdisciplinar Egas Moniz (CiiEM), Egas Moniz Cooperativa de Ensino Superior, Caparica, Portugal; c Hospital Garcia de Orta, Almada, Portugal; d Faculdade de Motricidade Humana – Universidade de Lisboa, Centro Interdisciplinar de Estudo da Performance Humana (CIPER), Lisboa, Portugal

## Abstract

**Introduction:**

The daily life of Chronic obstructive pulmonary disease (COPD) patients is characterised not only by chronic respiratory symptoms but also by exercise intolerance due to their breathlessness. Proper diagnosis and management of this disease consequently includes evaluation of exercise tolerance [1], frequently associated with a reduced functional exercise performance [2], thereby adversely affecting health status [3]. The purpose of this study is to analyse the impact of COPD on objectively-measured daily physical activity (DPA) through functional capacity and quality of life in these patients.

**Materials and methods:**

An observational study was performed with inclusion of seventy one men with moderate COPD (FEV1 54.6 ± 7.1%); age 63.8 ± 3.1 yrs; weight, 71.2 ± 8.3 kg; height, 169.0 ± 8.1 cm constituted the COPD group (COPDG), and 150 healthy subjects, age 64.2 ± 5.8 yrs; weight, 76.2 ± 11.3 kg; height, 169.8 ± 7.5 cm, were included as the healthy group – HG. The physical parameters assessed were strength, aerobic endurance, flexibility and agility/balance, by the Fullerton’s functional fitness tests. The health status was evaluated through the Medical Outcomes 36-item Short Form Health Survey (SF-36) questionnaire. The study was approved by the Ethics Committee of the Garcia de Orta Hospital and all participants gave their informed consent.

**Results:**

The values of the functional fitness test were significantly different (*p* < .05) between COPDG and HG groups for the following variables all expressed in mean ± SD: body mass index, 25.9 ± 3 vs 27.7 ± 4.1 kg.m2; 30-second chair stand 14.1 ± 1.7 vs. 18.2 ± 1.9 times; arm curl 15.7 ± 2.8 vs. 18.8 ± 4.9 times; 6-minute walk 498.8 ± 58.3 vs. 589.7 ± 88.6 m; 8-foot up-and-go 4.7 ± 0.8 vs. 5.1 ± 1 sec; chair sit-and-reach 0.81 ± 9.9 vs. −7.1 ± 10.6 cm respectively and no differences were observed for the back scratch test (DPOCG, −11.2 ± 9.7 cm and HG, −12.7 ± 11.6 cm). In health status DPOCG presented a significant decrease (*p* < .05) on perception of all domains of SF-36, except on body pain.

**Discussion and conclusions:**

In this study COPD patients have lower levels of functional capacity compared to healthy subjects. However, they were able to perform short tasks with higher speed. This trend was also evident in other study where COPD patients performed short term activities faster than healthy persons [4]. Limitation of activity and impaired quality of life are important outcomes of COPD and there is an association between physical activity and overall health status [5], which was also verified in this study. If functional capacity could be improved, by exercise training integrated in rehabilitation programs, probably we could also improve health status on these patients.
